# Oxidative Stress in Metabolic Disorders and Drug-Induced Injury: The Potential Role of Nrf2 and PPARs Activators

**DOI:** 10.1155/2017/2508909

**Published:** 2017-11-19

**Authors:** Ayman M. Mahmoud, M. Yvonne Alexander, Yusuf Tutar, Fiona L. Wilkinson, Alessandro Venditti

**Affiliations:** ^1^Physiology Division, Department of Zoology, Faculty of Science, Beni Suef University, Beni Suef, Egypt; ^2^Vascular Pathology Group, Centre for Biomedicine, School of Healthcare Science, Manchester Metropolitan University, Manchester, UK; ^3^University of Health Sciences, Mekteb-i Tıbbiye-i Şahane, Istanbul, Turkey; ^4^Department of Chemistry, Sapienza University of Rome, Rome, Italy

Oxidative stress plays a major role in metabolic disorders and a wide range of chronic diseases such as diabetes mellitus, obesity, metabolic syndrome, aging, cancer, osteoporosis, rheumatoid arthritis, cardiovascular diseases, and neurodegenerative disorders. In addition, drug-induced organ injury is well known to be associated with oxidative stress and inflammation. Considerable evidence indicates that oxidative stress and inflammation are the key pathophysiological processes underpinning these disorders. Therefore, modulation of oxidative stress represents an important strategy for the treatment of multiple human diseases.

The transcription factor nuclear factor erythroid 2 related factor 2 (Nrf2) is the master regulator of the basal and inducible expression of a large network of cytoprotective and antioxidant genes [1]. Under basal conditions, Nrf2 is bound to Kelch-like ECH-associated protein 1 (Keap1) which functions as a sensor protein against electrophiles and reactive oxygen species (ROS). Upon cell stimulation, Nrf2 dissociates from Keap1 and activated Nrf2 is translocated into the nucleus where it binds to the antioxidant response element (ARE) and leads to expression of target genes including heme oxygenase-1, NAD(P)H:quinone oxidoreductase 1, superoxide dismutase, catalase, glutathione peroxidase, and glutathione-S-transferase [2]. Thus, Nrf2 plays a role as a multiorgan protector against oxidative stress via inducing target genes. In recent years, Nrf2 has shown promise as a novel therapeutic target in diseases with underlying oxidative and inflammatory stress components [3–6].

Peroxisome proliferator-activated receptors (PPARs) are proteins that belong to the nuclear receptor family of ligand-activated transcription factors. The three main forms of peroxisome proliferator-activated receptors (PPAR*α*, PPAR*β*/*δ*, and PPAR*γ*) belong to a superfamily of nuclear receptors that function as transcription factors regulating the expression of multiple genes. Upon ligand binding, they form heterodimers with retinoid X receptor (RXR) and result in modulation of gene transcription [7]. PPARs regulate a variety of biological processes in various tissues. Among their effects, PPAR*α* controls lipid metabolism and inflammatory processes [8], PPAR*β*/*δ* regulates glucose utilization, cell differentiation, and inflammation [9], and PPAR*γ* is involved in adipocyte differentiation, glucose metabolism, and inflammatory pathways [10]. Upon activation, PPARs are known to exert anti-inflammatory and antioxidant properties via suppressing nuclear factor-*κ*B, decreasing ROS production, and upregulating the expression of antioxidant enzymes [11].

Recent reports point to coactivation and possible interaction between PPARs and Nrf2 through multiple mechanisms. Coactivation of PPAR*γ* and Nrf2 has been shown to protect against oxidative stress, inflammation, and carcinogenesis [4, 5, 12–14]. Ongoing and future research will probably provide efficient PPARs and Nrf2 modulating agents for preventing and treating metabolic and other common disorders.

This special issue encompasses cutting edge research and review articles focusing on the role of Nrf2 and PPARs in modulating oxidative stress and inflammation. It includes 8 novel research articles and 3 reviews describing the role of Nrf2 and PPARs in various pathological conditions, summarized as follows:

1. Drug-induced oxidative stress and hepatotoxicity

*Research article*: “Gamma-Glutamylcysteine Ethyl Ester Protects against Cyclophosphamide-Induced Liver Injury and Hematologic Alterations via Upregulation of PPAR*γ* and Attenuation of Oxidative Stress, Inflammation, and Apoptosis.” In this article, S. Alqahtani and A. M. Mahmoud introduced evidence demonstrating the involvement of PPAR*γ* in mediating the hepatoprotective effect of the synthetic glutathione precursor gamma-glutamylcysteine ethyl ester. Activation of PPAR*γ* resulted in enhancement of antioxidant defenses and attenuation of cyclophosphamide-induced oxidative stress, inflammation, and apoptosis.

*Review article*: “Collaborative Power of Nrf2 and PPAR*γ* Activators against Metabolic and Drug-Induced Oxidative Injury.” C. Lee reviewed the general features of PPAR*γ* and Nrf2 signaling pathways in the context of oxidative stress conditions. One of the main sections of this review was the role of natural and synthetic Nrf2 and PPAR*γ* activators and the crosstalk between Nrf2 and PPAR*γ* in alleviating drug-related oxidative stress and damage.

(2) Endocrine system and diabetes

*Research article*: “NRF2 Plays a Critical Role in Both Self and EGCG Protection against Diabetic Testicular Damage.” This study by C. Pan et al. aimed to evaluate the protective role of epigallocatechin gallate (EGCG) against diabetic testicular damage and addressed the requirement of Nrf2. Eight-week-old normal and diabetic male C57BL/6 wild-type and Nrf2 knockout mice were treated with EGCG for 24 weeks. Nrf2 knockout abrogated both self and EGCG protection against diabetes-induced testicular weight loss, reduction in spermatozoa count, apoptotic cell death, endoplasmic reticulum (ER) stress, inflammation, and oxidative damage. Therefore, this study provides evidence that Nrf2 plays a central role in mediating the protective effect of EGCG against diabetic-induced testicular damage.

*Research article*: “Activation of the Nrf2-Keap 1 Pathway in Short-Term Iodide Excess in Thyroid in Rats.” The effect of normal and high iodide intake on the antioxidative action of sulfredoxin (Srx) and peroxiredoxin 3 (Prx 3) via Nrf2-Keap 1 pathway has been investigated in the thyroid of rats. The expression of Srx and Prx 3 are known to be regulated via Nrf2. Srx is a member of the oxidoreductase family that contributes to cellular redox balance, and Prx 3 is a critical scavenger for mitochondrial ROS. The results showed that the activation of Nrf2 signaling, Srx, and Prx 3 may play a key role in protecting the thyroid gland from excess iodide-induced oxidative stress.

*Research article*: “Antioxidant Treatment Induces Hyperactivation of the HPA Axis by Upregulating ACTH Receptor in the Adrenal and Downregulating Glucocorticoid Receptors in the Pituitary.” J. P. Prevatto et al. tested the hypothesis that an imbalance in the redox system not only increases ROS production but also alters the homeostasis of the hypothalamus-pituitary-adrenal (HPA) axis culminating in its hyperactivation. The results showed activated HPA axis, increased levels of systemic glucocorticoids, decreased expression of Nrf2 and HO-1 in the pituitary, upregulated adrenocorticotropic hormone (ACTH) receptors in the adrenal gland, and downregulated glucocorticoid receptors in the pituitary. Therefore, the indiscriminate use of antioxidants may represent a risk to develop several morbidities related to persistent hypercorticoidism.

(3) Nonalcoholic steatohepatitis

*Research article*: “Genetic Nrf2 Overactivation Inhibits the Deleterious Effects Induced by Hepatocyte-Specific c-met Deletion during the Progression of NASH.” Based on the previous findings that overexpression of Nrf2 was able to reduce triglyceride accumulation and ROS production and suppress the levels of liver steatosis and fibrosis in c-met-deficient hepatocytes, P. Ramadori et al. provided *in vivo* evidence for the role of Nrf2 in preventing the deleterious effects induced by hepatocyte-specific c-met deletion during the progression of nonalcoholic steatohepatitis (NASH). In c-met/Keap1 knockout mice fed a methionine-choline-deficient (MCD) diet, Nrf2 overexpression reduced triglycerides accumulation, dampened the exacerbation of oxidative stress, drastically reduced the number of apoptotic cells, decreased the influx of infiltrating inflammatory cells, and attenuated the enhanced development of fibrosis.

(4) Hepatic encephalopathy

*Research article*: “*Commiphora molmol* Modulates Glutamate-Nitric Oxide-cGMP and Nrf2/ARE/HO-1 Pathways and Attenuates Oxidative Stress and Hematological Alterations in Hyperammonemic Rats.” In a rat model of hyperammonemia, a serious complication of liver disease which may lead to encephalopathy and death, A. M. Mahmoud et al. investigated the effect of *Commiphora molmol* resin extract on the glutamate-NO-cGMP and Nrf2/ARE/HO-1 signaling pathways. Activation of Nrf2 by *C. molmol* resin extract protected against excess ammonia via attenuation of oxidative stress and inflammation and modulation of the glutamate-NO-cGMP signaling pathway. In addition, *C. molmol* prevented hematological alterations and ameliorated both the activity and the expression of cerebral Na^+^/K^+^-ATPase and therefore might be a promising protective agent against hyperammonemia.

(5) Chronic kidney disease

*Research article*: “Expression of the *NRF2* Target Gene *NQO1* Is Enhanced in Mononuclear Cells in Human Chronic Kidney Disease.” Reduced Nrf2 activity has been reported in models of chronic kidney disease (CKD). In this study, J. Shen et al. quantified the NQO1 gene expression as a readout parameter for Nrf2 signaling in monocytes of patients with CKD with and without dialysis therapy. When compared to healthy control subjects, CKD patients showed an upregulated gene expression of Nrf2 and NQO1 and a slight increase in the NQO1 protein content in monocytes from these patients. The study concluded that Nrf2 activation in monocytes of CKD patients is modulated through an influence on both gene expression and protein content of Nrf2 targets in a complex way.

(6) Cardiovascular function and disease

*Review article*: “The Role of Nrf2 in Cardiovascular Function and Disease.” In this review article, S. Satta et al. summarized the mechanisms regulating the activity of Nrf2 and the role of Nrf2 in preventing mitochondrial dysfunction in cardiovascular disease. The authors highlight the central role of Nrf2 signaling in endothelial dysfunction, atherosclerosis, vascular calcification, hypertension, diabetic cardiomyopathy, and in the aging heart. In the last section of this review, the authors provide a summary of the role of Nrf2 activators in the treatment of cardiovascular disease.

*Research article*: “Probucol Protects Rats from Cardiac Dysfunction Induced by Oxidative Stress Following Cardiopulmonary Resuscitation.” The objective of this study was to investigate the protective effect of the lipid-lowering agent probucol on cardiac injury induced by cardiac arrest (CA) in rats. CA is one of the most critical cardiovascular phenomena. Probucol protected against CA in rats as evidenced by the improved restoration of spontaneous circulation (ROSC) rate, alleviated oxidative stress, prolonged survival time, and improved hemodynamic parameters, and cardiac function. These protective effects of probucol are mediated through activating Nrf2 signaling.

(7) Pregnancy disorders

*Review article*: “Modulatory Mechanism of Polyphenols and Nrf2 Signaling Pathway in LPS Challenged Pregnancy Disorders.” In this review article, T. Hussain et al. focused on the modulatory activity of flavonoids on oxidative stress-mediated pregnancy insults. They describe the role of Nrf2 activation in cases of pregnancy disorders.

The editors anticipate this special issue to be of interest to the readers and expect researchers to benefit in making further progress in the understanding of Nrf2 and PPARs activators.

## References

[1] Hayes J. D., Dinkova-Kostova A. T. (2014). The Nrf2 regulatory network provides an interface between redox and intermediary metabolism. *Trends in Biochemical Sciences*.

[2] Niture S. K., Khatri R., Jaiswal A. K. (2014). Regulation of Nrf2—an update. *Free Radical Biology & Medicine*.

[3] Mahmoud A. M., Wilkinson F. L., Jones A. M. (2017). A novel role for small molecule glycomimetics in the protection against lipid-induced endothelial dysfunction: involvement of Akt/eNOS and Nrf2/ARE signaling. *Biochimica et Biophysica Acta (BBA) - General Subjects*.

[4] Mahmoud A. M., Germoush M. O., Alotaibi M. F., Hussein O. E. (2017). Possible involvement of Nrf2 and PPAR*γ* up-regulation in the protective effect of umbelliferone against cyclophosphamide-induced hepatotoxicity. *Biomedicine & Pharmacotherapy*.

[5] Mahmoud A. M., Al Dera H. S. (2015). 18*β*-Glycyrrhetinic acid exerts protective effects against cyclophosphamide-induced hepatotoxicity: potential role of PPAR*γ* and Nrf2 upregulation. *Genes & Nutrition*.

[6] Kamel E. M., Mahmoud A. M., Ahmed S. A., Lamsabhi A. M. (2016). A phytochemical and computational study on flavonoids isolated from *Trifolium resupinatum* L. and their novel hepatoprotective activity. *Food & Function*.

[7] Michalik L., Wahli W. (2008). PPARs mediate lipid signaling in inflammation and cancer. *PPAR Research*.

[8] Guan Y., Zhang Y., Breyer M. D. (2002). The role of PPARs in the transcriptional control of cellular processes. *Drug News & Perspectives*.

[9] Tanaka T., Yamamoto J., Iwasaki S. (2003). Activation of peroxisome proliferator-activated receptor *δ* induces fatty acid *β*-oxidation in skeletal muscle and attenuates metabolic syndrome. *Proceedings of the National Academy of Sciences of the United States of America*.

[10] Tontonoz P., Spiegelman B. M. (2008). Fat and beyond: the diverse biology of PPAR*γ*. *Annual Review of Biochemistry*.

[11] Pascual G., Fong A. L., Ogawa S. (2005). A SUMOylation-dependent pathway mediates transrepression of inflammatory response genes by PPAR-*γ*. *Nature*.

[12] Mahmoud A. M., Hussein O. E., Hozayen W. G., Abd El-Twab S. M. (2017). Methotrexate hepatotoxicity is associated with oxidative stress, and down-regulation of PPAR*γ* and Nrf2: protective effect of 18*β*-glycyrrhetinic acid. *Chemico-Biological Interactions*.

[13] Mahmoud A. M., Hozayen W. G., Ramadan S. M. (2017). Berberine ameliorates methotrexate-induced liver injury by activating Nrf2/HO-1 pathway and PPAR*γ*, and suppressing oxidative stress and apoptosis in rats. *Biomedicine & Pharmacotherapy*.

[14] Mahmoud A. M., Mohammed H. M., Khadrawy S. M., Galaly S. R. (2017). Hesperidin protects against chemically induced hepatocarcinogenesis via modulation of Nrf2/ARE/HO-1, PPAR*γ* and TGF-*β*1/Smad3 signaling, and amelioration of oxidative stress and inflammation. *Chemico-Biological Interactions*.

